# A sensitive and specific point-of-care detection assay for Zaire Ebola virus

**DOI:** 10.1038/emi.2016.134

**Published:** 2017-01-18

**Authors:** Xiao-Ai Zhang, Sabrina Li, Jesus Ching, Hui-Ying Feng, Kun Yang, David L Dolinger, Long-Di Zhang, Pan-He Zhang, Wei Liu, Wu-Chun Cao

**Affiliations:** 1 State Key Laboratory of Pathogen and Biosecurity, Beijing Institute of Microbiology and Epidemiology, Beijing 10071, China; 2 Coyote Bioscience Company, Beijing 100085, China; 3 Coyote Bioscience Company, Campbell, WA 95008, USA; 4 Technology and Business Development Officer, Geneva 1211, Switzerland

**Dear Editor,**

The 2014–2015 West African Ebola virus disease (EVD) outbreak is caused by Ebola virus (EBOV, Zaire species); one out of five species in the EBOV genus, which causes a high fatality rate in infected humans (60%–90%).^1^ Since its initial identification in 2014 in Guinea, EVD has spread widely to the neighboring countries of Liberia and Sierra Leone, as well as to Europe and the United States, making the current outbreak by far the largest since its discovery in 1976. Under circumstances where no vaccine is available, effective EVD control measures are mainly dependent on early diagnosis and strict isolation of the patients who test positive with reliable and accurate diagnosis methods.

Although EBOV can be cultured, and antibody detection methods are available, real-time PCR with reverse transcription (RT–PCR) assays are currently widely used for EBOV laboratory diagnosis.^2, 3^ However, conventional real-time RT–PCR methods require an instrument with hands-on preparation to run an accurate test, which is less accessible in rural areas, causing critical delays in diagnoses and hampering control efforts.^4^ To minimize the spread of an outbreak, portable, accurate, simple point-of-care (POC) diagnostic tests that have high sensitivity (>93%) and specificity (>95%) are needed to detect the virus at the epicenter of the infection.^4, 5^ New advances in EBOV POC tests include enzyme-linked immunosorbent assay, RT–PCR assays, and biosensor-based EVD detection.^6, 7, 8, 9^ Real-time, portable genome sequencing for EVD surveillance was also reported.^10^ These diagnostic tools demonstrate effectiveness in identifying EVD cases.

In this study, we developed a real-time RT–PCR-based EBOV POC test (Coyote Bioscience Co., Ltd, Beijing, China) that can be performed without the need for RNA extraction and uses one-step real-time RT–PCR directly on any type of real-time PCR cycler. The entire process can be completed within 1.5 h, which is much quicker than the 3–4 h required for conventional real-time RT–PCR. In the 2014 EVD outbreak in Sierra Leone, the POC test was evaluated at the China Mobile Laboratory in Sierra Leone. Four hundred twenty-nine whole-blood samples from suspected individuals and 132 oropharyngeal swab samples from corpses were collected, with a waiver to provide written informed consent, during EVD outbreak under the agreement between the Sierra Leone and Chinese governments. All samples were inactivated at 56 °C for 1 h in a mobile BSL-3 laboratory and then analyzed simultaneously for EBOV by conventional real-time RT–PCR and POC tests (Supplementary Materials and Methods). For the conventional real-time RT–PCR test, viral RNA was isolated from samples using QIAamp Viral RNA Mini Kit (Qiagen, Germantown, MD, USA) under BSL-3 standards, and a one-step real-time RT–PCR kit (Shenzhen Puruikang Biotechnology Co., Ltd, Shenzhen, China) was used to detect EBOV in a PCR lab. The positive and negative predictive values of the kit compared with real-time RT–PCR with primers and probes recommended by the WHO were 98.9% and 100%, respectively. The limit of detection of the kit was 5.0 × 10^2^ copies/mL. The kit is specific for Zaire EBOV and has no cross-reactivity for other species of EBOV and 22 pathogens. The POC test was performed in a portable POC setting (Mini8 Real-time PCR system; Coyote Bioscience) in a PCR laboratory. The limit of detection of the POC test was 5.0 × 10^2^ copies/mL. The test is specific for Zaire EBOV, and no cross reaction was observed with other species of EBOV or dengue virus, chikungunya virus, yellow fever virus and severe fever with thrombocytopenia syndrome bunyavirus. The Mini8 Real-Time PCR System is 205 × 190 × 98 mm (length × width × height) and weighs 2.1 kg. The system is compatible with a 12 V DC power supply and can be operated with the supplied battery pack and treated with UV light for disinfection. Eight samples can be tested in a single run on this system. Personal protective equipment and operation procedures were adopted according to Laboratory diagnosis of EVD from the World Health Organization (WHO) (http://www.who.int/csr/resources/publications/ebola/laboratory-guidance/en/). The results in conventional real-time RT–PCR test were compared with those obtained with POC tests.

Out of the 429 blood samples analyzed, identical results between POC and conventional real-time RT–PCR tests were observed for 424 samples. Two samples gave false-negative results, and three samples yielded false-positive results for the POC test (Supplementary Figure S1). The sensitivity and specificity of the POC test compared with conventional real-time RT–PCR test were 99.3% (95% confidence interval (CI), 97.5%–99.9%) and 97.9% (95% CI, 94.0%–99.8%), respectively (Table 1). Out of the 132 swab samples analyzed, identical results between POC and conventional real-time RT–PCR tests were observed for 130 samples. Two samples yielded false-negative results, and no false-positive results were observed using the POC test. The sensitivity and specificity of POC test compared with conventional real-time RT–PCR test were 97.3% (95% CI, 90.5%–99.7%) and 100% (95% CI, 93.9%–100%), respectively (Table 1). In overall samples, the sensitivity and specificity of POC test compared with conventional real-time RT–PCR test were 98.9% (95% CI, 97.1%–99.7%) and 98.5% (95% CI, 95.7%–99.7%), respectively (Table 1). Given the limited conditions during the EVD outbreak, we made no further assessment of discordant results by alternative assays. However, all seven discordant results had high cycle threshold values by two tests (Supplementary Figure S2). The range of viral loads of the conventional RT–PCR for the 554 samples tested was 5.3 × 10^2^–5.0 × 10^7^ copies/mL. Both sensitivity and positive predictive values of the POC test compared with conventional real-time RT–PCR test were 100.0% from patients who had greater than 1.0 × 10^5^ copies/mL of EBOV (Supplementary Table S1). For 355 EVD patients positive for both the POC test and the conventional real-time RT–PCR test, the cycle threshold values of the two tests were positively correlated in both blood (*P*<0.001, Supplementary Figure S3A) and swab samples (*P*<0.001, Supplementary Figure S3B).

Our data suggest that the POC test is highly sensitive and specific and performs well for EVD detection. The POC test showed several advantages compared with the conventional real-time RT–PCR test. Firstly, the POC test requires no prior sample processing and involves a simple one-step real-time RT–PCR by adding only three ingredients, making it operable by minimally trained technicians. In addition, the simple operation of the POC test could reduce the opportunity for nosocomial infection of laboratory staff and contamination during the experiments. Another advantage of the POC test is the small input volume of specimens, making it possible to be applied for fingerstick blood samples.

EBOV has been classified as a Category-A critical biological agent, with the potential to be used as a bioterrorism agent, highlighting the need for its accurate and timely detection in variable sample types. The WHO recommends repeat testing of symptomatic patients when negative results by RT–PCR were obtained <three days after disease onset.^11^ The consequences of false-negative results of laboratory diagnosis could be dire to outbreak management, especially during early disease stages. Compared with conventional real-time RT–PCR, the POC test has minimized the time to obtain a result, thereby allowing clinicians or public health staff to make expeditious decisions. In addition, EVD patients with high viral loads posed an increased risk of developing fatal outcome and transmitting the virus to close contacts, thus demanding closer vigilance.^12, 13^ The conventional real-time RT–PCR test was time-consuming and cost-prohibitive. Therefore, in an effort to maintain a sustained monitor of the viral load, the POC test might also offer an advantage. Even in medical centers with well-developed laboratory tests, the POC test could also serve as a valuable supplement when diagnostic laboratories are overwhelmed with testing requests once a pandemic occurs.

We also demonstrated that the POC test had comparable diagnostic accuracy to the conventional real-time RT–PCR test for oropharyngeal swab samples. The analysis based on 132 swab samples gave results similar to those of the whole-blood samples. Together with the results of earlier studies, our results indicate that swab specimens are highly sensitive for diagnostic testing of corpses.^14^

A limitation of the study is that no fingerstick blood samples were collected to perform the POC test. Venous blood sampling requires trained medical personnel, bears high risk for nosocomial infection and is especially difficult to perform with newborns and infants or adult patients who reject venipuncture due to cultural and religious beliefs.

In summary, the combined application of the POC test with the help of POC equipment gave highly sensitive and specific results in detecting EBOV in outbreak events.

## Figures and Tables

**Table 1:** Performance of point-of-care test versus conventional real-time RT–PCR test for Ebola virus detection

	**Point-of-care test**		
	**Blood (*n*=429)**	**Swab (*n*=132)**	**All (*n*=561)**
Prevalence (% of patients that tested positive by conventional real-time RT–PCR test)	286/429 (66.7%)	73/132 (55.3%)	359/561 (64.0%)
Sensitivity	284/286 (99.3%, 97.5%–99.9%)	71/73 (97.3%, 90.5%–99.7%)	355/359 (98.9%, 97.1%–99.7%)
Specificity	140/143 (97.9%, 94.0%–99.6%)	59/59 (100%, 93.9%–100%)	199/202 (98.5%, 95.7%–99.7%)
Negative predictive value	140/142 (98.6%, 95.0%–99.8%)	59/61 (96.7%, 88.7%–99.6%)	199/203 (98.0%, 95.0%–99.5%)
Positive predictive value	284/287 (99.0%, 97.0%–99.8%)	71/71 (100%, 94.9%–100%)	355/358 (99.2%, 97.6%–99.8%)

Abbreviations: confidence interval, CI; PCR with reverse transcription, RT–PCR.

Data are *n/N* (%) or *n/N* (%, 95% CI).
