# Supplementary material for: A sensitive and specific point-of-care detection assay for Zaire Ebola virus
Source: Emerg Microbes Infect. 2017 Jan 18;6(1):e5–. doi: 10.1038/emi.2016.134 (PMC5285498; doi:10.1038/emi.2016.134)
Supplement: Supplementary Table S1 [file emi2016134x1.docx]

| **Conventional real-time RT-PCR test** | **Point-of-care test** | |
| --- | --- | --- |
|  | **Sensitivity (95% CI)** | **Positive predictive value (95% CI)** |
| Blood viral load |  |  |
| ≥10^7^ (n = 8) | 100.0% (63.1-100) | 100.0% (63.1-100) |
| 10^7^~10^6^ (n = 38) | 100.0% (90.7-100) | 100.0% (90.7-100) |
| 10^6^~10^5^ (n = 81) | 100.0% (95.5-100) | 100.0% (95.5-100) |
| <10^5^ (n = 159) | 98.7% (95.5-99.8) | 100.0% (97.7-100) |
| Swab viral load |  |  |
| ≥10^7^ (n = 1) | 100.0% (2.5-100) | 100.0% (2.5-100) |
| 10^7^~10^6^ (n = 3) | 100%(29.2-100) | 100%(29.2-100) |
| 10^6^~10^5^ (n = 3) | 100%(29.2-100) | 100%(29.2-100) |
| <10^5^ (n = 66) | 97.0%(89.5-99.6) | 100.0% (94.4-100) |

**Supplementary Table S1 Performance of the Ebola virus point-of-care test versus conventional real-time RT-PCR test by Ebola viral load**

Data are n/N (%) or n/N (%, 95% CI).
