# Supplementary material for: A sensitive and specific point-of-care detection assay for Zaire Ebola virus
Source: Emerg Microbes Infect. 2017 Jan 18;6(1):e5–. doi: 10.1038/emi.2016.134 (PMC5285498; doi:10.1038/emi.2016.134)
Supplement: Supplementary Materials and Methods [file emi2016134x2.docx]

**Supplemental Materials and Methods**

**Conventional real-time RT-PCR test for Ebola virus (EBOV)**

Viral RNA was isolated from 140 uL of whole blood or swab samples using QIAamp Viral RNA Mini Kit (Qiagen, Germantown, MD, USA) according to the manufacturer’s instructions. Detection Kit for EBOV RNA (PCR-Fluorescence Probing) (Shenzhen Puruikang Biotechnology Co., Ltd., Shenzhen, China) was used according to the manufacturer’s instructions in a volume of 25 µL containing 20 µL of PCR Buffer A, 2 µL of PCR Buffer B, and 3 µL of total RNA. PCR was carried with one cycle of 42ºC for 5 min and 94ºC for 10 sec, followed by 40 cycles of 94ºC for 5 sec and 55ºC for 30 sec in a LighterCycler 480 Real-Time PCR platform (Roche Applied Science, USA). Positive and negative controls were included. The kit is specific for Zaire EBOV and has no cross-reactivity for other species of EBOV and 22 pathogens (dengue virus, severe fever with thrombocytopenia syndrome virus, influenza virus A, coronavirus, hantavirus, yellow fever virus, chikungunya virus, encephalitis virus B, measles virus, forest encephalitis virus, herpes simplex virus, HIV virus, Xinjiang hemorrhagic fever virus, yersinia pestis, vibrio cholerae 0139, legionella pneumophila, burkholderia malei ATCC 23344, Neisseria meningitidis, bacillus anthracis, streptococcus pneumonia, salmonella typhi, shigella dysenteriae).

**EBOV point-of-care (POC) test**

The EBOV POC test (Coyote Bioscience Co., Ltd, Beijing, China) was performed in a POC setting (Mini8 Real-time PCR system; Coyote Bioscience). The FAM-labeled probe was specific for Zaire EBOV. The primers and TaqMan probes used for the EBOV detection at NP gene were as follows: 5′-GAGCATGGTCTTTTCCCTCA-3′ (forward primer), 5′-TCGCGAGACTCTGCATATTG-3′ (reverse primer), FAM5′-CGCCACAGCACACGGGAGT-BHQ1-3′ (probe). The ROX-labeled probe was specific for internal reference gene. The primers and TaqMan probes used for the internal reference gene were as follows: 5′-AGATTTGGACCTGCGTAGCG-3′ (forward primer), 5′-GAGCGGCTGTCTCCACAAGT-3′ (reverse primer), ROX5′-TTCTGACCTGAAGGCTCTGCGCG-BHQ2-3' (probe). POC test diagnosis using cut-off cycle threshold (CT) values for positive, equivocal, and negative of <30, 30.1–35, and >35, respectively. The POC test reacion was performed in a final volume of 30μL containing a one-fold final concentration of PCR mastermix in One-step Detection Kit and 2μL of eight fold diluted (the buffer was provided with the kit) blood or 10μL of throat swab solution. Forward and reverse primers were used at concentration of 200 nM, and the concentration of probe was 200 nM. The PCR conditions comprised a reverse transcriptional step of 1 minutes at 42 °C, followed by 10 cycles of 5 sec denaturation at 95 °C, and 10 sec extension at 45 °C, and then denaturation step of 1 minutes at 95 °C, this was followed by 40 cycles of 5 sec denaturation at 95 °C and 10 sec extension at 55 °C. Positive and negative controls were included. Real time data was analyzed using the Mini8 Real-Time PCR System software v.1.0 (Coyote Bio). The total time of detection is about 1.5 h.

**EBOV concentration determination**.

EBOV concentration was also determined with primers and probes used for EBOV detection using conventional real-time RT-PCR test. In brief, plasmids containing known copy number of amplification targets were included in conventional real-time RT-PCR test to generate a standard curve for quantification of test samples respectively. Positive and negative controls were included. The copy number of virus was determined by comparison with a serially diluted plasmid standard of known concentration.
