# Supplementary figures and images for: A sensitive and specific point-of-care detection assay for Zaire Ebola virus
Source: Emerg Microbes Infect. 2017 Jan 18;6(1):e5–. doi: 10.1038/emi.2016.134 (PMC5285498; doi:10.1038/emi.2016.134)

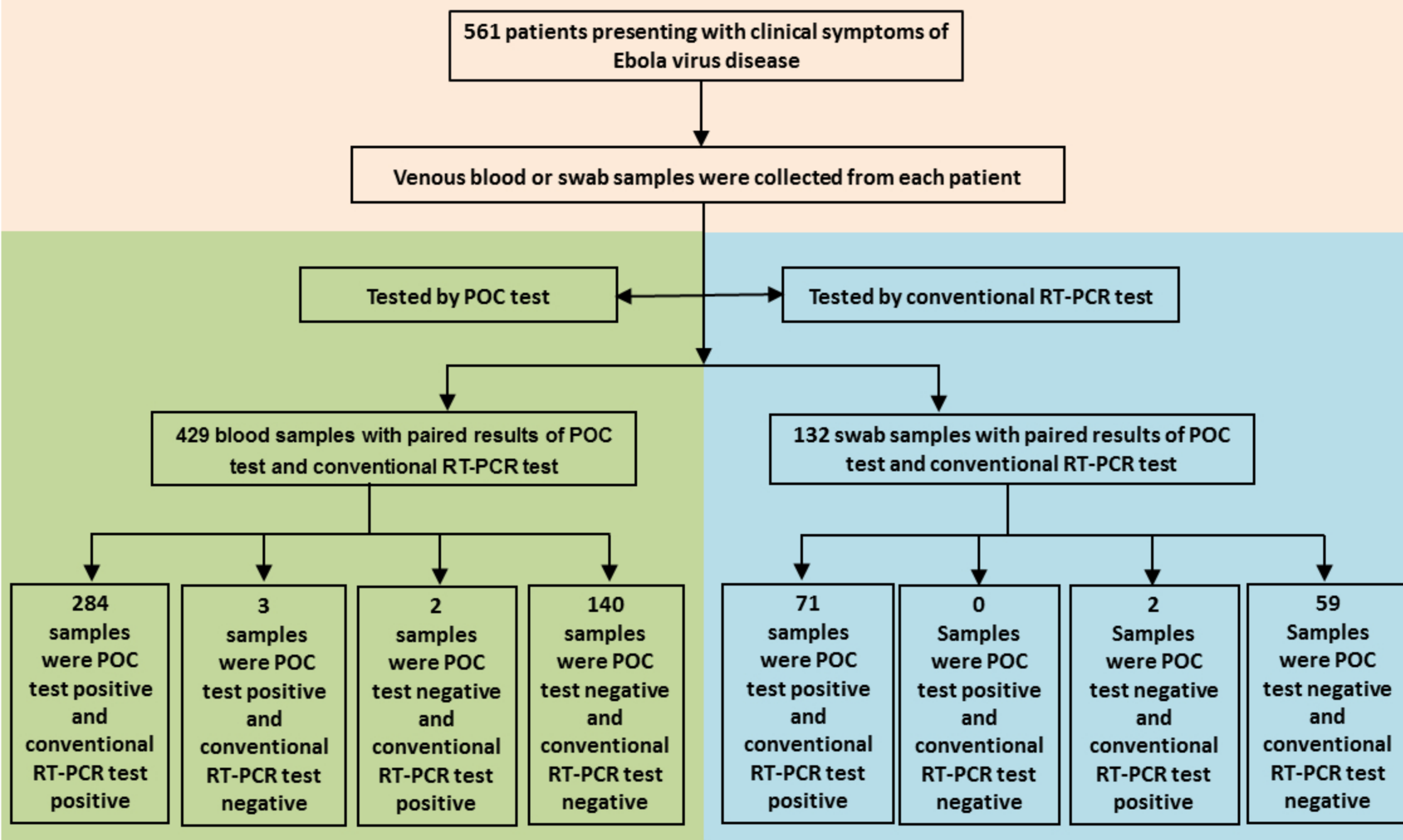

Supplementary Figure S1 Study profile for point-of-care test of Ebola virus disease.

Supplement: Supplementary Figure S1 [file emi2016134x3.pdf]

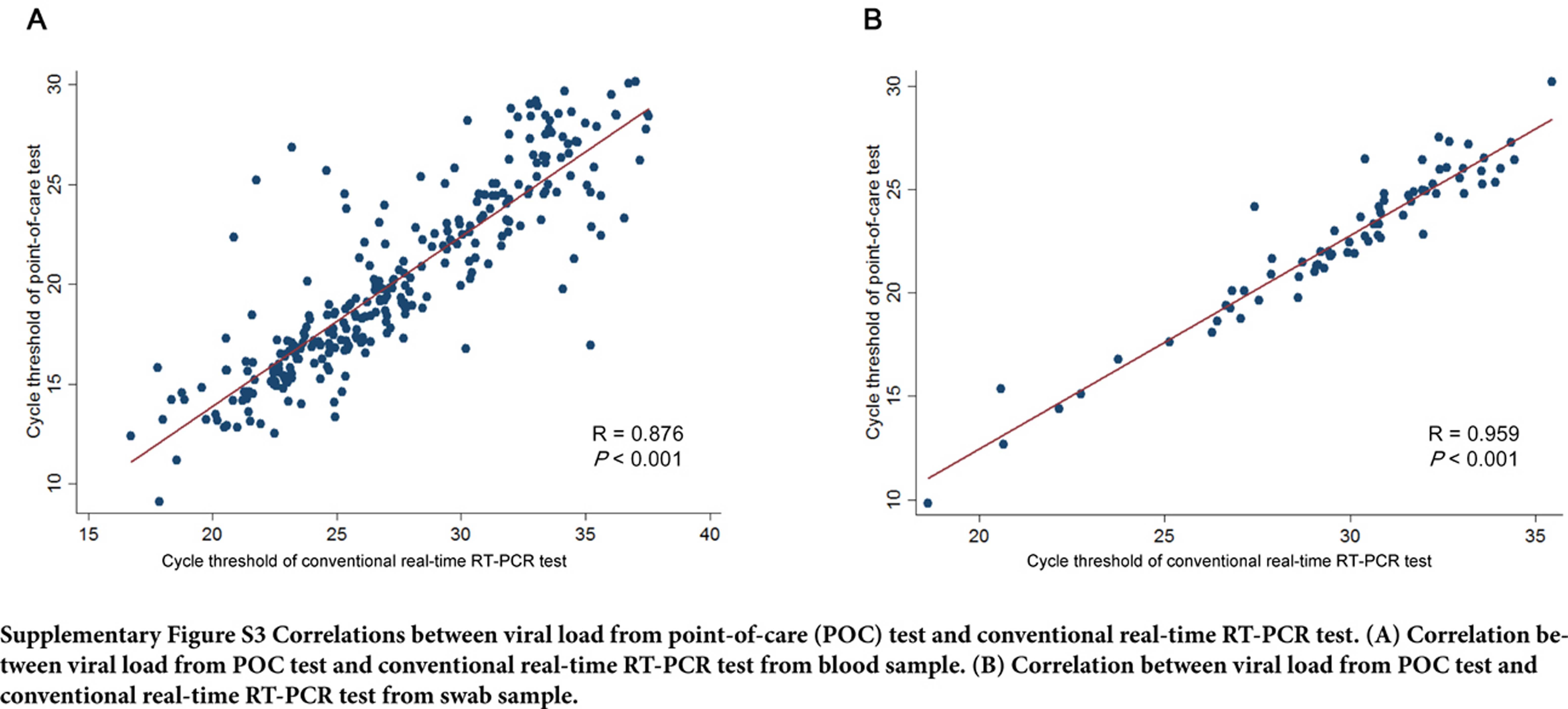

Supplement: Supplementary Figure S3 [file emi2016134x5.tif]
