# Supplementary material for: A sensitive and specific point-of-care detection assay for Zaire Ebola virus
Source: Emerg Microbes Infect. 2017 Jan 18;6(1):e5–. doi: 10.1038/emi.2016.134 (PMC5285498; doi:10.1038/emi.2016.134)
Supplement: Supplementary Figure S2 [file emi2016134x4.pdf]

A

Conventional real-time RT-PCR test		Point-of-care test	
**Result**	**Cycle threshold**	**Result**	**Cycle threshold**
Positive	34.26	Negative	NA
Positive	36.75	Negative	NA
Negative	NA	Positive	25.4
Negative	NA	Positive	23.82
Negative	NA	Positive	20.35

B

Conventional real-time RT-PCR test		Point-of-care test	
**Result**	**Cycle threshold**	**Result**	**Cycle threshold**
Positive	35.4	Negative	NA
Positive	34.57	Negative	NA

Supplementary Figure S2 Discordant results for Ebola virus (EBOV) between conventional real-time RT-PCR test and point-of-care (POC) test. (A) Five blood samples with discordant results of EBOV (conventional real-time RT-PCR test positive and POC test negative, $n = 2$; conventional real-time RT-PCR test negative and POC test positive, $n = 3$). (B) Two swab samples with discordant results of EBOV (conventional real-time RT-PCR test positive and POC test negative, $n = 2$).
