# Supplementary material for: A sensitive and specific point-of-care detection assay for Zaire Ebola virus
Source: Emerg Microbes Infect. 2017 Jan 18;6(1):e5–. doi: 10.1038/emi.2016.134 (PMC5285498; doi:10.1038/emi.2016.134)
Supplement: Supplementary Information [file emi2016134x6.pdf]

COYOTE

Mini8 Plus Real-Time PCR System

Version：EN / 20160413

Coyote, highly specialized in research & development, offers integrate molecular diagnostic solutions by providing devices from sample pre to gene amplification / detection.

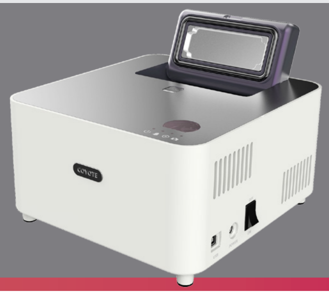

Coyote Bioscience Co., Ltd.
Tel: 010-64844237
Fax: 010-64844237-8000
Add: Room A211/213, 2/F, Shangdixinxilu#12 Haidian District, Beijing, China. 100085

www.coyotebio.com

COYOTE

Contents

Chapter 1 — Overview

Introduction

Chapter 2 — Setup

Unpack the Mini8 Plus System

Place Mini8 Plus on the Bench

Connect Mini8 Plus

Install the Mini8 Plus Software

Turn on the Mini8 Plus System

Chapter 3 — Workflow

Mini8 Plus System Workflow

Load the reaction tubes

Define a New Experiment

Set Up the Thermal Profile

Define the Plate Layout

Monitor Run

Data Analysis

Chapter 4 — System Information

Lights

Specifications and Environmental Requirements

Electromagnetic Compatibility

Cleaning and Maintenance

Chapter 5 — Concepts

Overview

Introduction

Coyote Mini8 Plus Real-Time PCR System is designed for molecular diagnostic market.

It has a small footprint, with high detection sensitivity. The machine has two channels, one is SYBR/FAM, and the other is Texas Red / ROX. The sample capacity is 8 × 0.2mL PCR tubes, and it is compatible with any commercial SYBR® or TaqMan® qPCR kits.

The user interface on the computer is friendly and very simple to operate.

The fluorescence dye preference can be customized. This product requires 12V DC power supply, that is compatible with a car charger or a battery pack.

Coyote Bio engaged on designing and manufacturing innovative personal devices for both life science and molecular diagnosis. All our devices were vested portable features for field applications with 12V DC power or batteries while all redundant features were minimized and the key functions were developed more powerful and user-friendly. Therefore Coyote's devices are easy to handle and set, means that the labor cost could be significantly reduced.

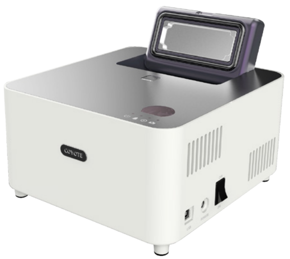

Service: Coyote Bioscience Co., Ltd.

Registered Address: 509, 510, 5th floor, Chuangyezhonglu#36, Haidian, Beijing

ZIP: 100085

TEL: 010-64844237

FAX: 010-64844237-8000

SALES: sales@coyotebio.com

Setup

Unpack the Mini8 Plus System

1. Lift the Mini8 Plus instrument out of the package. Place it on a flat surface and remove the packaging materials (Keep the box and packaging in case of a return).

2. Check the packing list as follows to ensure that all components are present and intact. Your system comes with:

A. Mini8 Plus instrument

B. Power adapter

C. Power cable

D. USB cable

E. DVD

F. User's manual

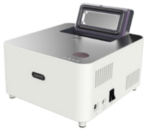

A

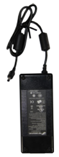

B

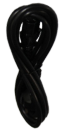

C

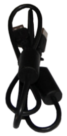

D

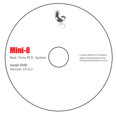

E

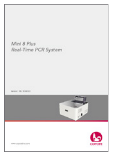

F

Place Mini8 Plus on the Bench

Benefit from its mini size, two distances of only 10cm (4 inches) for each sides of left and right are enough for ventilation, and also a distance of 5cm (2 inches) above is necessary for opening the lid.

Connect Mini8 Plus

1. Connect one connector of the USB cable to the port on the computer. Connect the other connector to the port on the side panel of the Mini8 Plus.

2. Connect the Mini8 Plus power cable to the DC power inlet on the side panel, and then to the wall outlet.

3. Plug the computer power cable to the wall outlet.

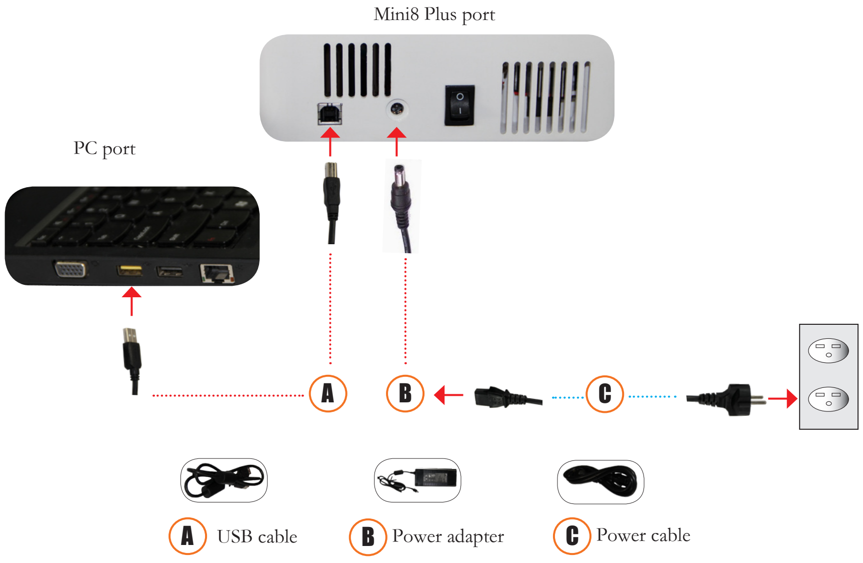

Install Mini8 Plus software

- Connect Mini8 Plus to computer and switch on the device;
- Load CD or open the software file, click setup program.

1) Before installation the Prerequisites Wizard must be started running to set up the environment as follow:

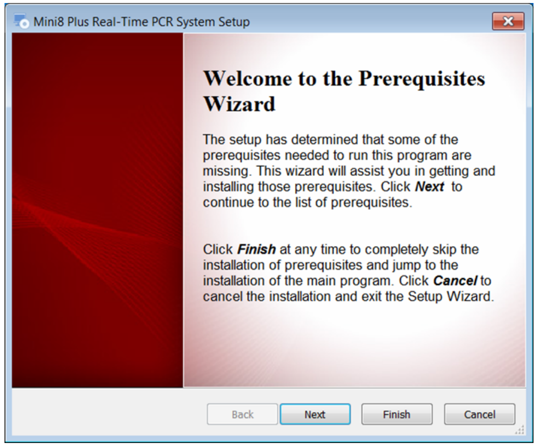

Click Next

Select 'DriverInstaller Application', '.NET Framework 4.0', 'NI LabVIEW Runtime 2014 SP1 f3' and Click next. Click'next' per dialog box continuously to install 'DriverInstaller Application', '.NET Framework 4.0', 'NI LabVIEW Runtime 2014 SP1 f3'.

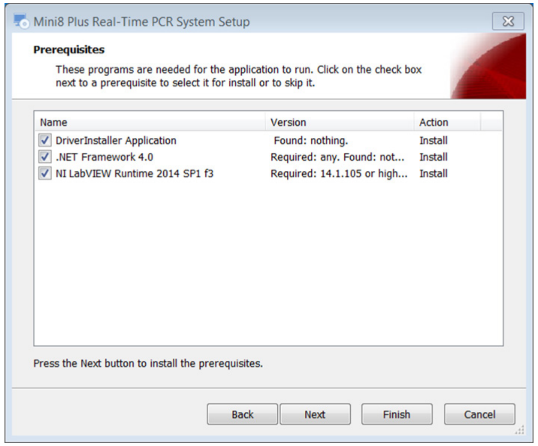

Click Next

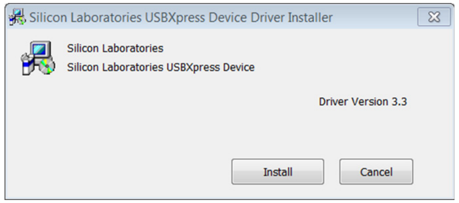

Click Install

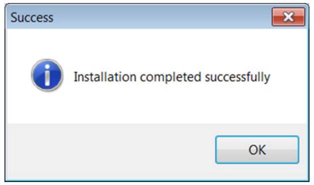

Click OK

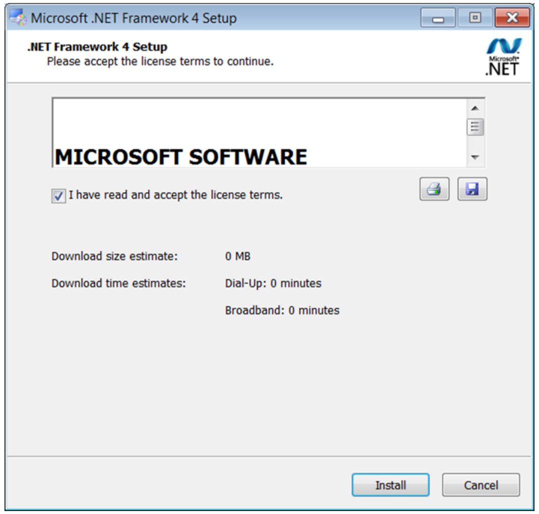

Click Install

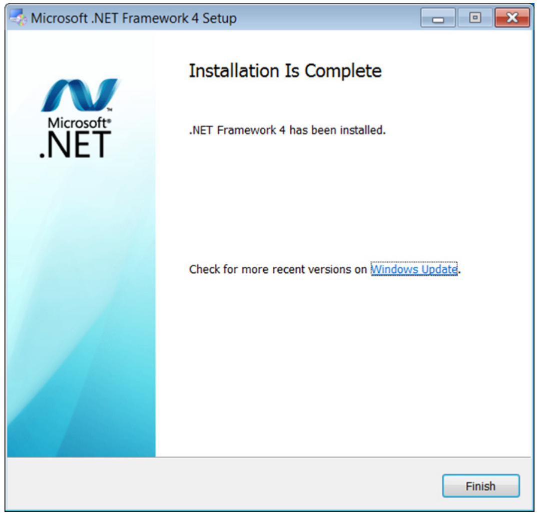

Click Finish

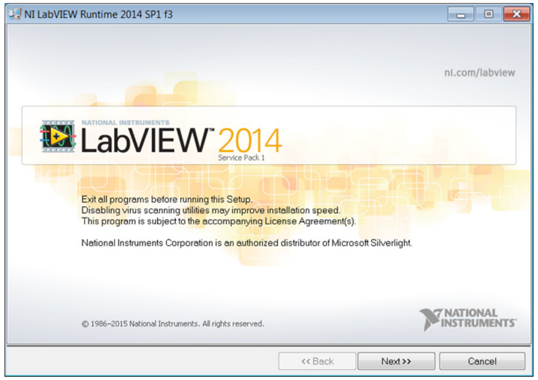

Click Next

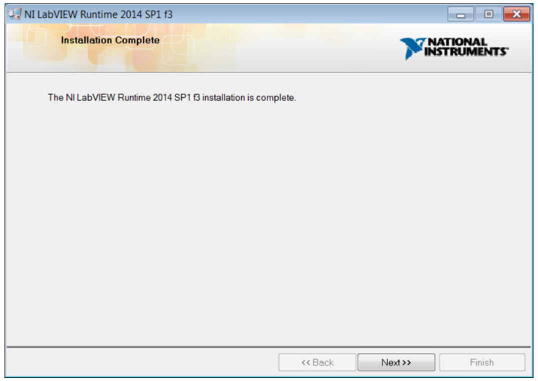

Click Next

• The System will automatically detect the operating environment, and check the installation options, If a project has been installed, you can manually cancel.

• Click next to start the installation.

Note: During the installation, the system may be requested to restart, you can choose to restart later.

2) The software installation will be boot up once the environment detection completed.

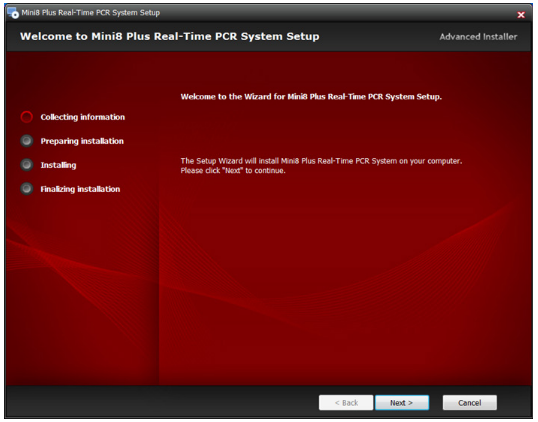

Click Next

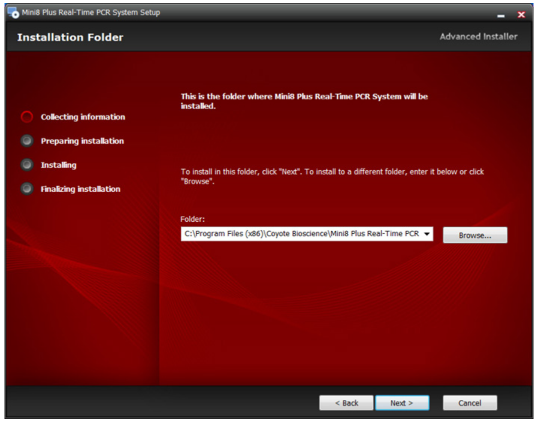

Select installation path and click Next to continue

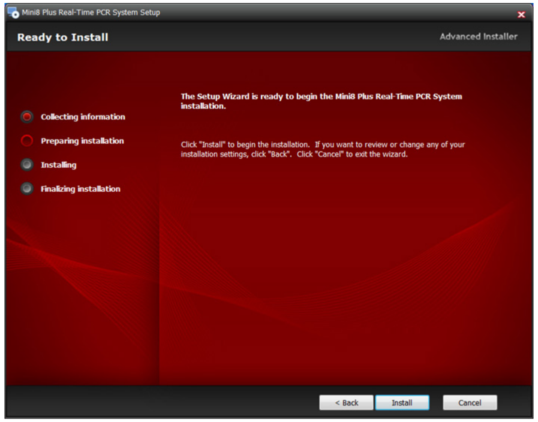

Click Install to start the installation

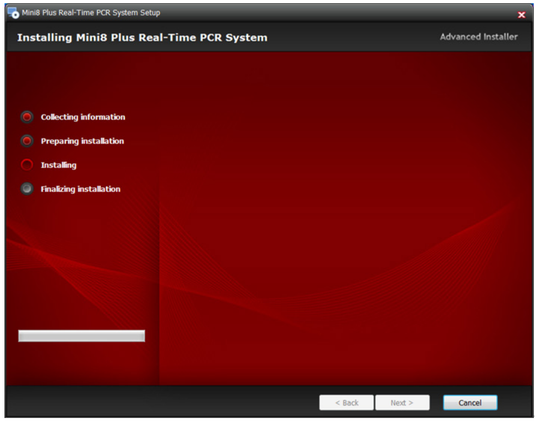

The Mini8 Plus software is installing

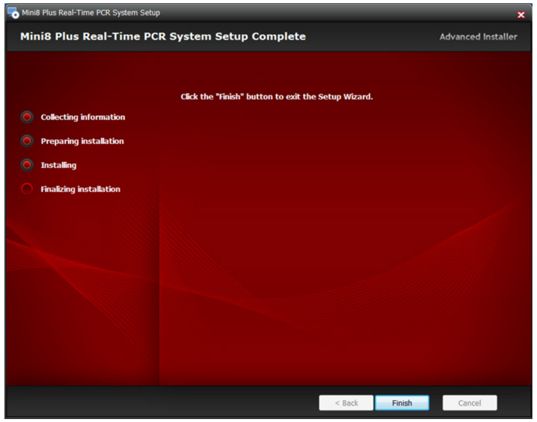

After the installation click Finish to close the setup program

Turn on the Mini8 Plus System

Turn on the Mini8 Plus instrument, then double-click the Mini8 Plus icon 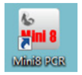 to start the software.

*When the Power indicator lights on the front panel stop flashing and remain solid, the instrument is ready.

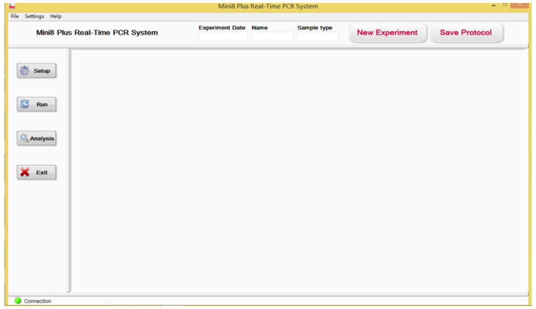

Workflow

Mini8 Plus System Workflow

1. Prepare the sample strip, load it into the Mini8 Plus, and close the lid.

2. Double-click the Mini8 Plus icon 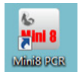 on desktop to open the software.

3. Define and name the experiment, save the experiment.

Tip: to use a pre-defined thermal profile and plate layout for your experiment, click 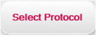

and select one of the template experiments saved in your computer.

4. Review the thermal profile and adapt it if needed.

5. Set up the plate layout by defining assays, samples, and standards and assigning them to wells.

6. Start the run. The Monitor Run tab opens.(Do not open the lid while a run is in progress.This will corrupt the data.)

7. When the run is completed, open the Mini8 Plus lid. Remove the strip from the block. Dispose of any hazardous materials into appropriate containers for biohazard, caustic materials, according to your local safety regulations.

Load reaction tubes

1. Thaw all necessary reagents (templates, primers, probes, and master mix).

2. Turn on the computer, then the Mini8 Plus, and wait until the Mini8 Plus "Power" light is flashing 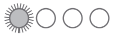 (Standby mode)

3. Confirm that the block and optical path are clear of visible contaminants and there is no physical damage to the system.

4. Pipette samples and qPCR reagents into the strip according to your protocol. (Warning: Wear protective gloves and eyewear when operating with any material that might be considered caustic or hazardous.)

5. Open the Mini8 Plus lid and place the strip on the dock.

6. Close the Mini8 Plus lid.

7. Proceed to define a new experiment.

Warning

Do not touch hot lid. The hot lid temperature would be up to 105°C (221°F) when the device is working .

Define a New Experiment

1. Double-click the Mini8 Plus icon on the desktop to open the software.

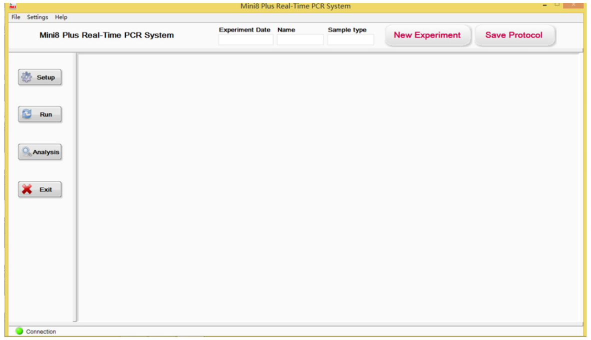

2. Click "New Experiment", the New Experiment tab opens, enter an experiment name and sample type.

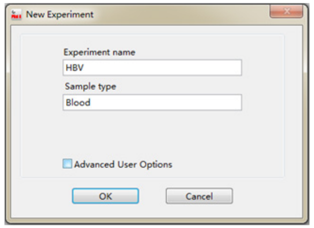

Experiment Date	Name	Sample type
2016/3/22 1:20	HBV	Blood

Advanced user options (optional functions)

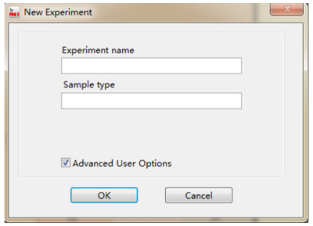

Select advanced user options and click OK

1) If you want to pause running when ΔRn reaches the setting value, please input terminal ΔRn for each sample. This function can help you to get the PCR amplification product in the status you want.

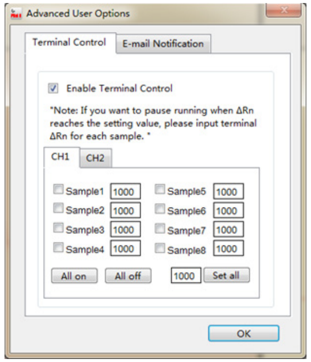

2) Fill in your Email, when the running is over, the result report (Excel file) will be sent to your email.

PS. The computer need to be connected to the network.

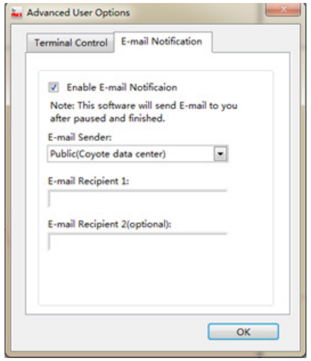

Set up the Thermal Profile

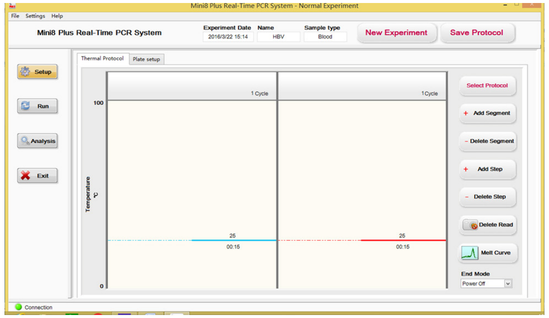

1. Click **Setup** , the Setup window opens, with the Thermal Protocol tab visible.

2. Click **+ Add Segment** / **– Delete Segment** to add/delete segment.

3. Click **+ Add Step** / **– Delete Step** to add/delete step.

4. The camera icon 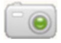 indicates that the fluorescence is being read.

5. Click **Melt Curve** to add the Melt Curve profile.

6. Click **Save Protocol** to save Protocol **Protocol data (*.pdt)**

7. Click **Select Protocol** to choose and use the saved Protocol (*.pdt)

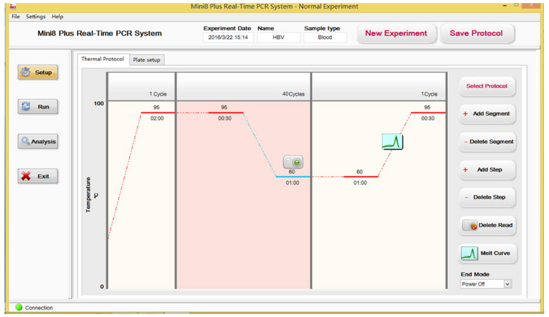

Define the Plate Layout

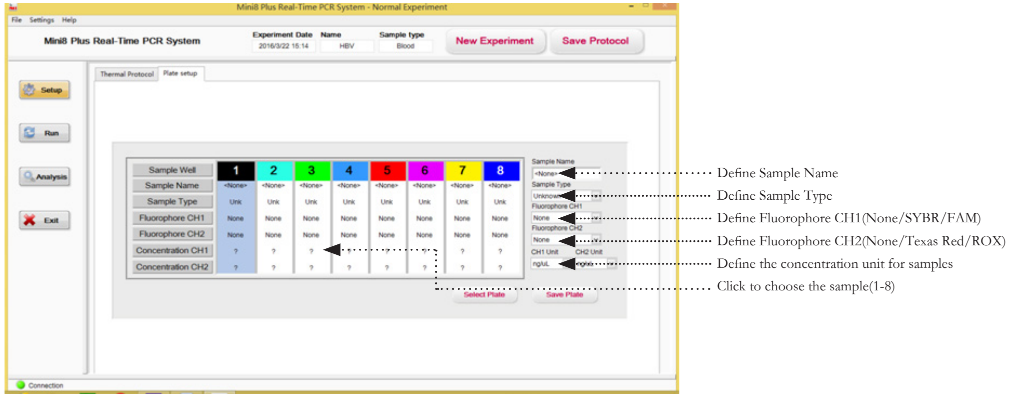

The "plate setup" involves the following steps:

1. Click **Plate setup** , the Plate Setup window opens.

2. Click **1 2 3 4 5 6 7 8** to choose the sample.

3. Set up sample name.

4. Set up sample type: unknown, positive control, negative control, standard.

When the sample type is defined as "Standard" **Sample Type: Standard**

the concentration setting interface will be promoted for user to set the "Standard" concentration in the following interfaces:

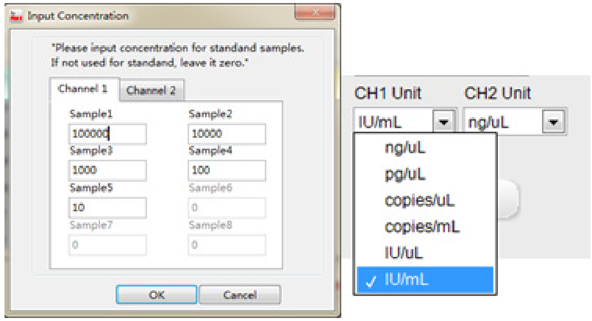

5. Set up fluorophore CH1(None/SYBR/FAM)

6. Set up fluorophore CH2(None/Texas Red/ROX)*

7. Click **Save Plate** to save plate layout

8. Click **Select Plate** to choose and use the saved plate

Monitor Run

Click **Run** to enter the running interface, and click **START** to start the running.

Click **PAUSE** to pause the running.

Click **STOP** to stop the running.

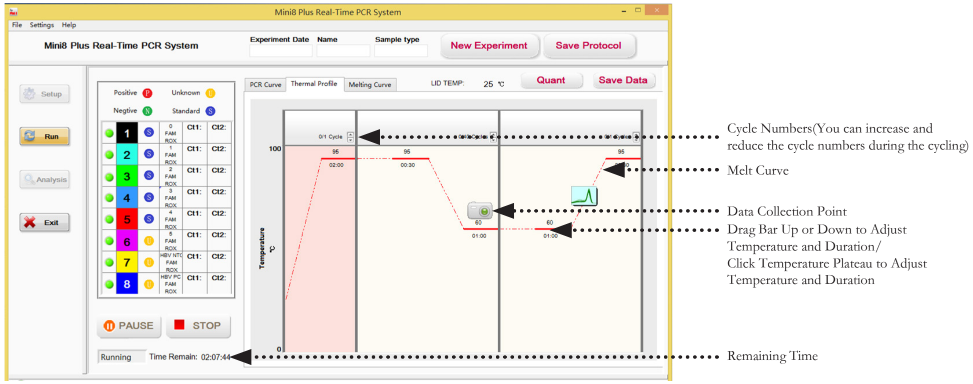

The Real time PCR curve will be showed during the amplification.

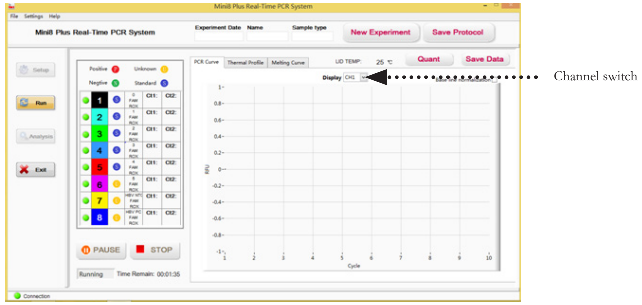

Warning

Do not open the lid while a run is in progress. It may allow extraneous light enter the system so the data will be corrupted.

Note

If you do not set up the fluorophore channel when set the thermal profile, there will be not any curve showing neither during nor after the process of amplification, for the fluorescence will not be read.

When the running is over, click **Save Data** to save data(*.edt).

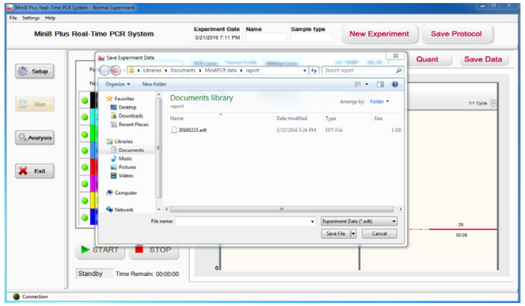

Data Analysis

Click 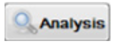

to show the saved data.

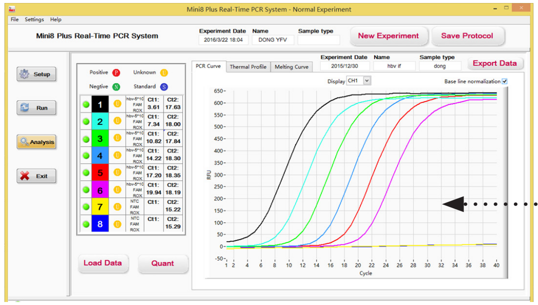

PCR amplification curve

CH1 FAM

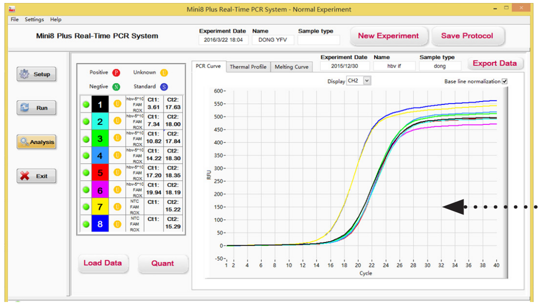

PCR amplification curve

CH2 ROX

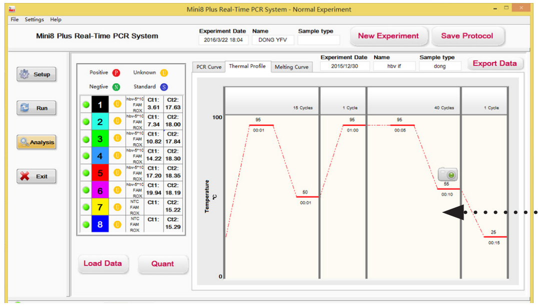

Thermal Profile

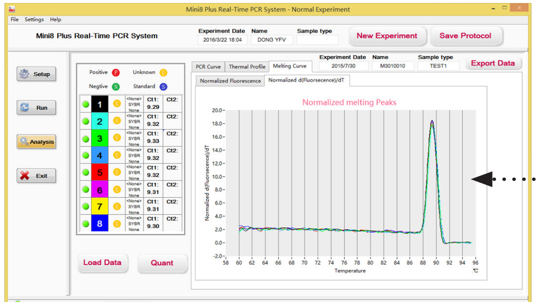

Melt Peak

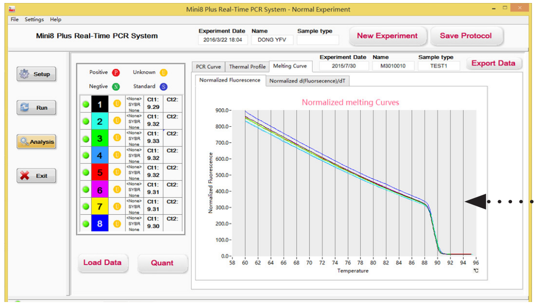

Melt Curve

Click **Quant** to show the quantification result.

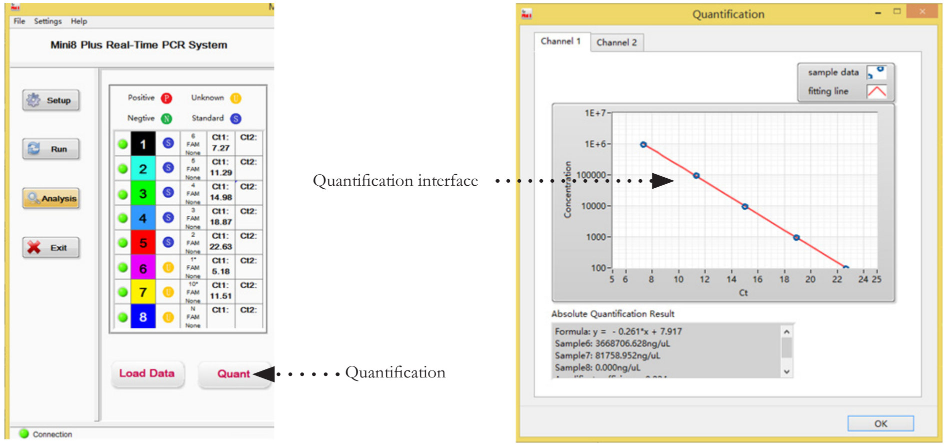

Click **Export Data** to export the result report (*.Excel).

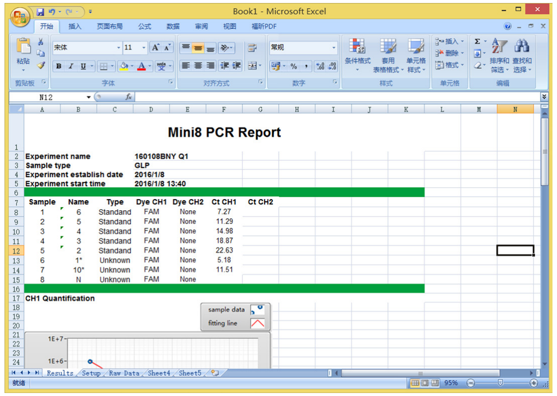

And then click **Exit** to exit the software. Turn off the Mini8 Plus instrument.

System Information

Lights

The Mini8 Plus System has four indicator lights on the bottom right corner of the top panel: Power, Error, Status, and Scaning . The following table shows the meaning of each combination of off ,on,and flashing lights.

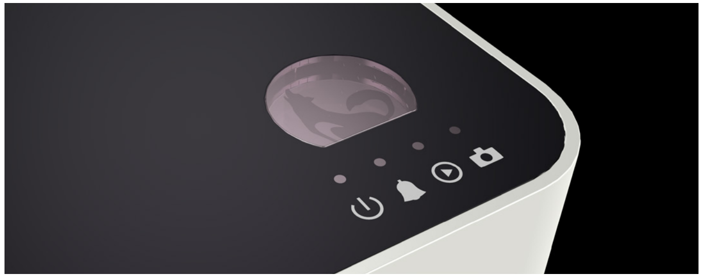

Lights	Description
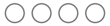	Power off
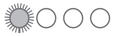	Standby mode
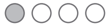	Power on
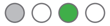	PCR is running
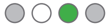	PCR is running & scanning
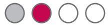	Fatal Error (instrument might be overheated)

System Information

Specifications and Environmental Requirements

Optical	Light Source	High Power LED
	Detector	Photodiodes
Thermal	Heating/cooling module	Peltier
	Ramping Rate (Max.)	3°C/s
	Thermal Uniformity	±0.2°C
	Thermal Accuracy	±0.2°C
	Temperature Range	4-100°C
Operational	Sample Capacity	8 wells
	Reaction Volume	15-150μL
	Warm Up Time	1min
	Sensitivity of Detection	1 copy
	Melt Curve Resolution	Supported Resolution to 0.5°C
	Multiplexing	Detect up to 2 dyes simultaneously, (FAM/ROX)
Physical	Dimensions	205×190×98 mm (L×W×H)
	Weight	2.1 kg
	Power	12V, 10A
Computer Requirement	System	WIN 7; WIN 8.1; WIN 10
Environmental	Environmental Temperature Range	Operating: 15°C to 30°C<br>Storage: 10°C to 60°C
	Environmental Humidity Range	Operating: 15-90% relative Humidity<br>Storage: 5-95% relative Humidity

Electromagnetic Compatibility

- To confirm proper operation: The electromagnetic environment should be evaluated prior to operation of the system.
- Do not use this system in close proximity to sources of strong electromagnetic radiation (e.g. unshielded intentional RF sources), as these may interfere with proper operation.
- If you notice any interference, discontinue using the system until all issues are resolved. Resolution may include moving cords from other equipment away from the system, plugging the system into an outlet on a different circuit from other equipment, or moving the system away from other equipment. If you still have difficulties, contact COYOTE.

Cleaning and Maintenance

Clean the block and housing as needed, following these directions.

Caution: If hazardous or biohazardous materials are spilled onto or into the equipment, clean it immediately.

1. Turn the system off and allow the block to cool completely.

2. Using a lint-free cloth slightly dampened with clean water, gently wipe

the surfaces of the equipment. If a stronger cleaning agent is needed, use a lint-free cloth slightly dampened with 95% isopropyl alcohol.

Follow these practices for regular maintenance of your Mini8 Plus system.

1. Every time before using the system, visually check it to confirm there is no obvious physical damage such as dents, frayed cords, or damaged levers. If you see any damage, discontinue using and contact COYOTE Technical Support.

2. Once a year, run a known test sample to confirm accurate analysis.

Concepts

• The weight of one genome (g) = (size of genome in bp) x (618 g/mol/bp)/Avogadro's number

One human genome (g) = $(3x10^{9}bp) \times (618g/mol/bp) / (6.02x10^{23}) = 3.08x10^{-12}g$

One haploid cell (sperm/egg) = 3.08 pg of DNA

One diploid cell = 6.16 pg of DNA

• RNA concentration (µg/µl) = (A260×40×D)/1000, where D = dilution factor and A260= absorbance at 260 nm.

• DNA concentration (µg/µl) = (A260×50×D)/1000, where D = dilution factor and A260= absorbance at 260 nm.
